# Relief of Lumbar Symptoms After Cervical Decompression in Patients with Tandem Spinal Stenosis Presenting with Primarily Lumbar Pain

**DOI:** 10.7759/cureus.940

**Published:** 2016-12-24

**Authors:** Daniel R Felbaum, Islam Fayed, Jeffrey J Stewart, Faheem A Sandhu

**Affiliations:** 1 Neurosurgery, Medstar Georgetown University Hospital; 2 Georgetown University School of Medicine, Georgetown University

**Keywords:** tandem spinal stenosis, cervical stenosis, lumbar stenosis, cervical myelopathy, spine

## Abstract

Objective: Tandem cervical and lumbar spinal stenosis (TSS) is classically described as intermittent claudication, gait disturbance, and clinical findings of mixed myelopathy and polyradiculopathy. Rarely, patients can present with TSS manifesting in isolated lumbar pain. Several reports have demonstrated improved lumbar back pain and radiculopathy after decompressive cervical spine procedures. We present six patients with dramatic resolution of lumbar spine related symptoms after decompression of the cervical spinal cord despite presenting solely with lower back complaints.

Methods: Clinical records of the senior author (F.A.S.) gathered from April 2006 to March 2013 were retrospectively reviewed identifying six patients presenting solely with lumbar symptoms and diagnosed with TSS based on history and physical examination.

Results: Six patients with a mean age of 55 (range 39 to 60) presented with solely lower back symptoms and clinical findings suspicious for TSS. Mean follow-up time for all patients was 12 months (range three to 27 months, median 11.5 months). Three patients underwent a cervical procedure as the principal operation, while the remainder had the lumbar spine decompressed initially. All patients that underwent a cervical procedure initially experienced a dramatic decrease or complete resolution of their preoperative lower back pain and radiculopathy (mean preoperative VAS of 6.7 vs. 3.7 postoperative). The remainder of patients with persistent lumbar symptoms resolved after a subsequent cervical operation.

Conclusion: Patients presenting with lumbar symptoms out of proportion to imaging require further investigation. We highlight the resolution of lumbar symptoms after a cervical procedure in a select group of patients presenting with lone lower back complaints. In patients presenting with symptoms disproportionate to lumbar imaging, treatment of cervical pathology may provide robust long-term relief of the initial lumbar-related presentation.

## Introduction

Degenerative spondylosis and stenosis are common conditions in the elderly population that typically manifest as a result of the degenerative changes associated with aging and stress that cause progressive encroachment on the spinal canal. Spinal stenosis is defined as a critical narrowing of the sagittal diameter of the spinal canal (<10 mm for cervical stenosis and <11 mm for lumbar stenosis), and patient presentation depends on this progressive narrowing [1]. It can occur at any level of the spine, but most often occurs in the more mobile cervical and lumbar spine [2]. Cervical stenosis typically presents with myelopathy, including gait disturbance, hyperreflexia, and weakness, while lumbar stenosis is associated with radiculopathy and neurogenic claudication.

Tandem spinal stenosis (TSS) was first described in 1964 and is defined as a critical narrowing of both the cervical and lumbar spine [3]. The incidence ranges from five percent to 25%, with a preponderance of cases in males greater than 50 years old [4-7]. The diagnosis of TSS may be elusive due to its varied presentation. Patients may present with a triad of intermittent neurogenic claudication, gait disturbance, and clinical findings of mixed myelopathy and polyradiculopathy in both the upper and lower extremities [5]. However, symptoms referable to either the cervical or lumbar spine can predominate or vary in onset [7]. Decompression of either the cervical, lumbar or both regions may lead to improvement in the associated symptoms of both conditions. As such, this mixed clinical presentation often perplexes clinicians as to the underlying spinal pathology, and the appropriate course of treatment remains under debate. While most patients with TSS undergo decompression of both cervical and lumbar regions, improvement of lumbar back pain and radiculopathy after decompression of the cervical spine has been reported [8].

In the present study, we identified six patients presenting with a chief complaint of severe low back pain. While these patients’ lumbar spine-related complaints dominated their initial clinical picture, radiographic and clinically significant cervical stenosis was subsequently identified based on directly elicited history and physical findings. All of these patients experienced dramatic resolution of back and lower extremity symptoms after decompression of their cervical stenosis. We highlight that, in a certain patient population with isolated but disproportionate lumbar symptoms, identification and treatment of cervical stenosis may provide long-lasting relief of these symptoms. Although this may appear to be categorized as tandem stenosis, we postulate that these patients fall into a separate group.

## Materials and methods

Clinical records of the senior author (F.A.S.) from April 2006 to March 2013 were retrospectively reviewed. Six patients were identified who presented with isolated lumbar symptoms, but either radiographic findings did not clearly correlate with their symptoms, or they had findings of myelopathy on physical examination which led to subsequent cervical imaging and identification of cervical stenosis. All preoperative spinal imaging (magnetic resonance imaging (MRI), computed tomography (CT) myelogram, or radiographs) were reviewed by an independent radiologist and the senior author. The degree of stenosis was graded based on cross-sectional area of the spinal canal at the worst area of stenosis [9]. All patients were assessed with preoperative and postoperative comprehensive neurologic exams and visual analog scale (VAS) pain scores. Informed consent was obtained from all the patients for this study.

## Results

Six patients with a mean age of 55 (range 39 to 60) presented with isolated lumbar symptoms and clinical findings suspicious for cervical stenosis that met our study criteria. Mean follow-up time for all patients was 12 months (range three to 27 months, median 11.5 months). Three patients had a history of prior lumbar surgery without sustained improvement of their back and/or lower extremity symptoms. Five patients underwent a cervical procedure that resulted in a dramatic decrease or complete resolution of their preoperative lower back pain and radiculopathy (Table 1). Using an unpaired two-tailed t-test, the mean preoperative low back VAS pain score of 6.7 improved postoperatively to 3.7 in a statistically significant manner (p = .05).

**Table 1:** Procedures and VAS scores

Age	Sex	Preoperative VAS	Cervical Procedure	Postoperative VAS	Myelopathy	Signal Change	Prior Lumbar Surgery
60	M	8	C6 corpectomy	1	Yes	Yes	No
59	F	7	C4-6 corpectomy	2	Yes	Yes	No
60	M	8	C3-7 laminoplasty	9	Yes	No	No
57	M	8	C3-4 corpectomy	5	Yes	No	Yes
57	M	1	C5-6 ACDF	0	Yes	No	Yes
39	M	8	C3-6 ACDF	5	Yes	No	Yes

## Discussion

### Case illustrations

Case 1

**Figure 1:**
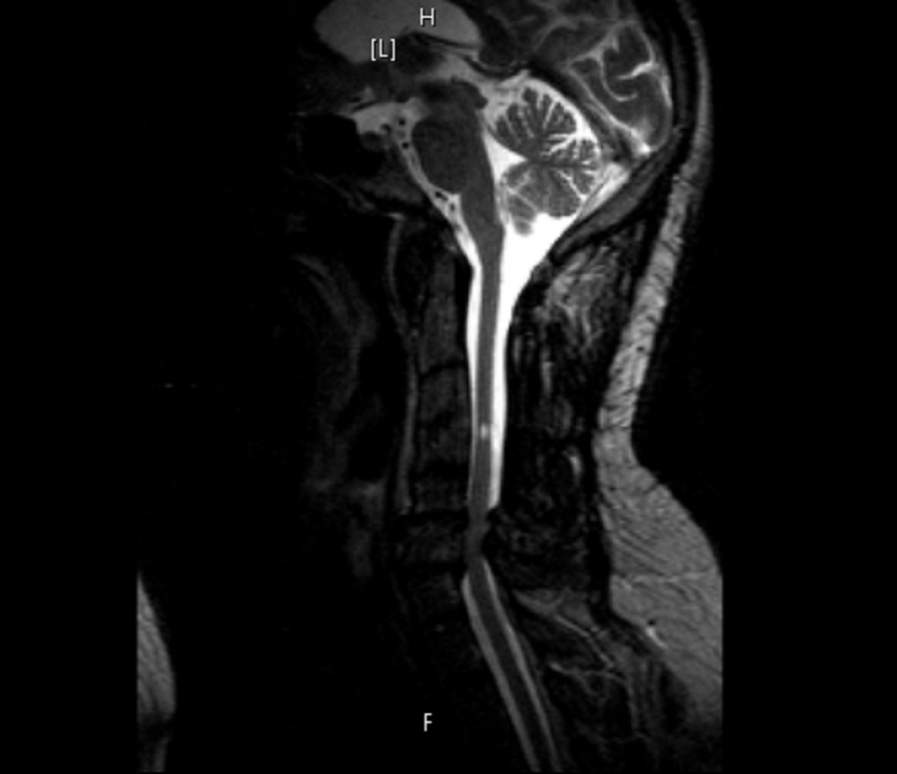
(Case 1) Preoperative sagittal MRI of cervical spine

**Figure 2:**
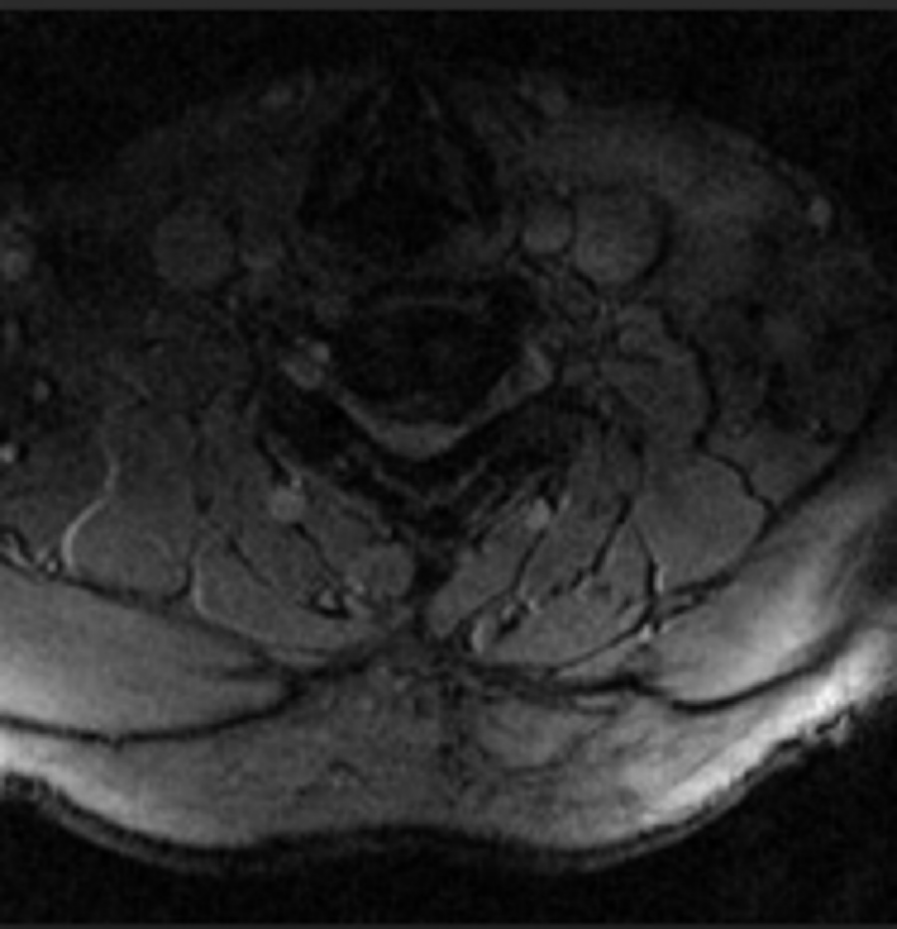
(Case 1) Preoperative axial MRI of cervical spine at C5/6

A 60-year-old man presented with severe refractory low back pain and pain radiating to both lower extremities. VAS pain score was eight out of 10. Upon further examination, he was noted to have significant hyperreflexia and spasticity of both upper and lower extremities, with positive Hoffman’s sign bilaterally. Magnetic resonance imaging (MRI) showed disc herniations at C5-6 and C6-7 with severe stenosis posterior to the C6 vertebral body (Figures 1-2). In contrast, imaging of his lumbar spine was unimpressive, revealing a mild disc bulge at L3-4. Given the degree of hyperreflexia and spasticity, in addition to the significant cervical spinal stenosis, cervical decompression was elected. The patient underwent a C6 corpectomy without complication. In the following months the patient’s preoperative urinary retention, hand weakness, and low back pain with radiating leg pain almost completely resolved, with the patient reporting a VAS of 1. As a result, no further intervention was deemed necessary.

Case 2

**Figure 3:**
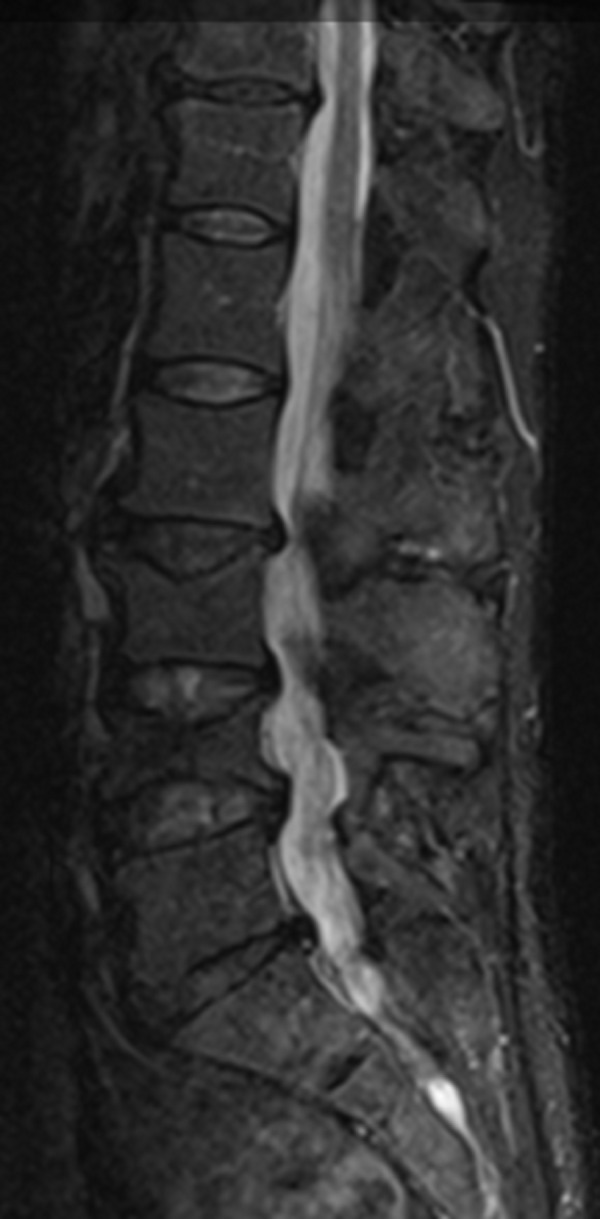
(Case 2): Preoperative sagittal MRI of the lumbar spine

**Figure 4:**
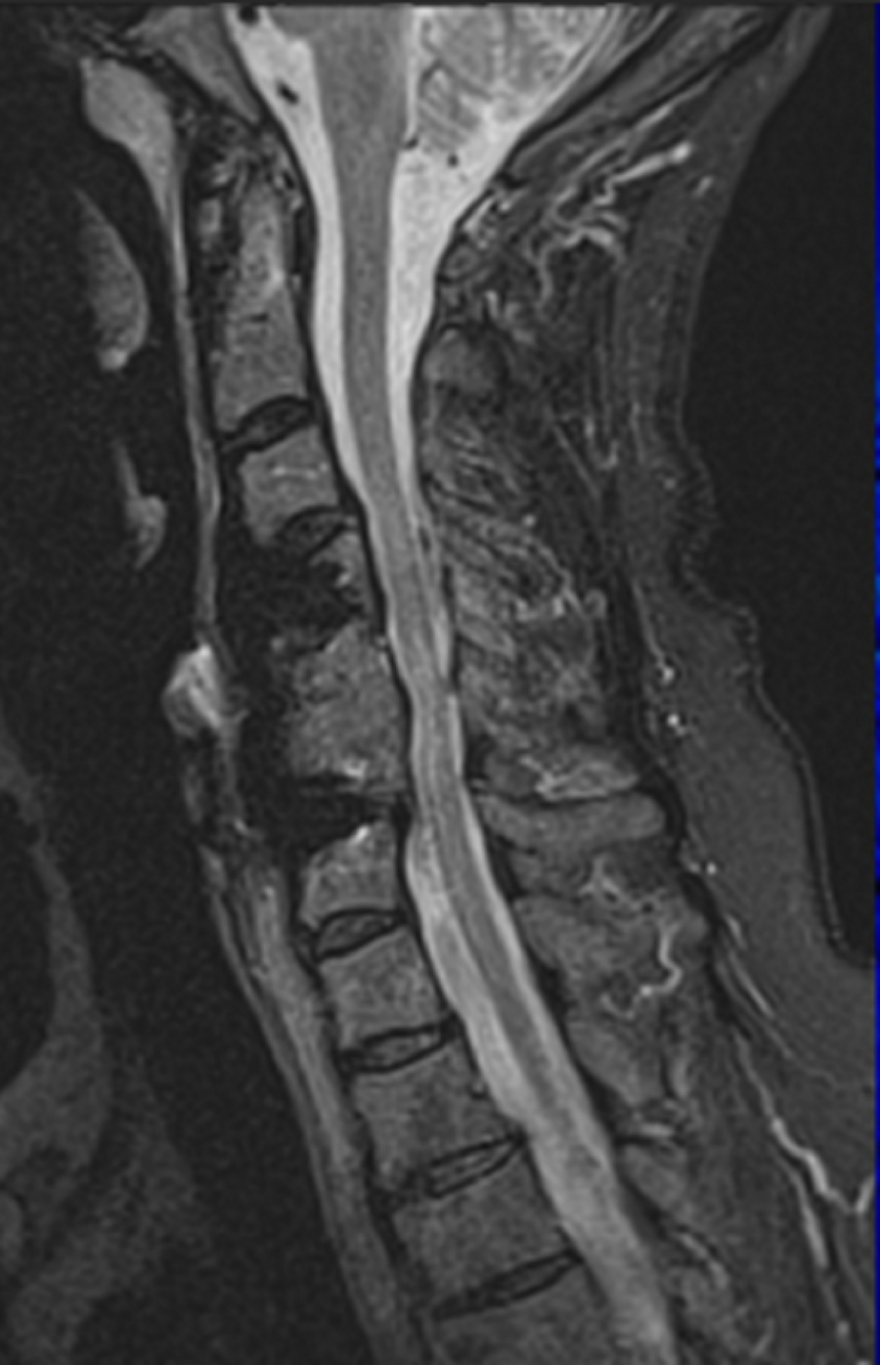
(Case 2) Preoperative sagittal MRI of the cervical spine

**Figure 5:**
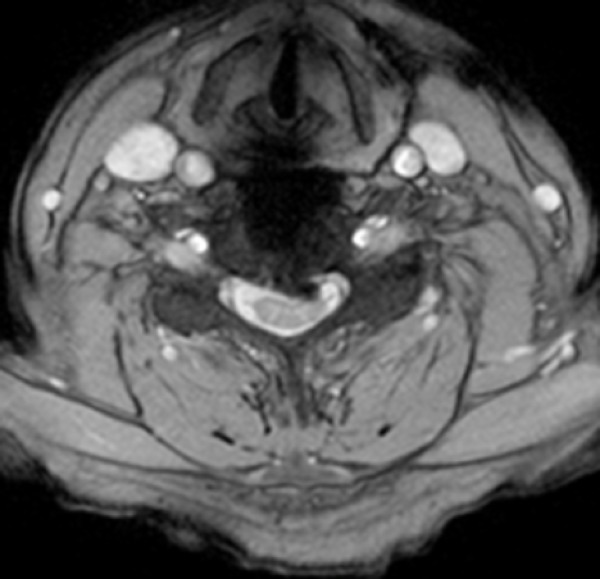
(Case 2) Preoperative axial MRI of the cervical spine

A 59-year-old woman presented with a chief complaint of progressively worsening severe low back pain. Her past surgical history was significant for a prior anterior cervical discectomy and fusion. Her preoperative VAS score for back pain was seven out of 10. On exam, her strength was grossly full, but she was noted to be severely myelopathic. She was diffusely hyperreflexic, with positive Hoffman’s sign and sustained clonus. She had a wide-based antalgic gait. These findings were only identified on physical exam and detailed history. Radiographic workup demonstrated multilevel lumbar spondylosis with both central and foraminal stenosis (Figure 3). Due to her myelopathic symptoms and exam findings disproportionate to her presenting complaints, an MRI of her cervical spine was performed. Imaging demonstrated severe cord compression with signal change at the level of her prior C4-6 anterior cervical discectomy and fusion (Figures 4-5). Because of the severe cord compression and signs of myelopathy, the patient underwent a C4-6 corpectomy with C3-7 posterior instrumentation and fusion with no complications. During the postoperative period, her VAS score for low back pain decreased to two. The patient was pleased with the resolution of her neck and low back pain. Her only remaining symptoms were residual weakness of the left deltoid and biceps. Again, no further surgical intervention was deemed necessary.

### Discussion

Patients with TSS do not typically present with a chief complaint isolated to lumbar spine pathology. Instead, it is most often characterized by a triad of intermittent neurogenic claudication, gait disturbance, and combined upper and lower motor neuron findings [10]. TSS may also mimic several upper motor neuron disorders, but, importantly, it must be differentiated due to its amenability to surgical treatment. This series attempts to highlight that there is a subset of TSS patients that present primarily with lumbar spine associated symptoms. Cervical stenosis with evidence of myelopathy is only identified upon further interrogation and clinical examination. Furthermore, treatment of only the cervical stenosis may completely ameliorate the chief presenting symptom of low back pain.

The mechanism of TSS is hypothesized due to cervical spinal cord compression that results in referred pain caused by irritation of the spinothalamic tract and/or interruption of descending raphe nuclei projections that serve to modulate ascending nociceptive pathways. Furthermore, due to the somatotopic organization of upper motor neurons from the motor cortex to their respective nerve roots, compression via cervical stenosis may result in a mixture of upper and lower motor neuron findings [11]. This may explain the dramatic improvement in low back pain and radiculopathy observed after cervical decompression seen in this series.

The controversial nature of the treatment process for TSS can be seen in many cases in which the secondary condition becomes more evident after surgical treatment of the primary condition [1,6,12]. Some authors suggest that the order of surgical decompression should be determined by the location of the more severe symptoms and recommend deferring to cervical decompression in cases that are unclear [1,6,13]. Our findings support this recommendation, as all three patients that initially underwent lumbar decompression required subsequent cervical decompression. More importantly, the chief presenting complaint of low back pain significantly improved after a cervical operation. In our small series of patients, decompressing the cervical spine was crucial in providing clinically significant relief of preoperative complaints. This is further highlighted in the patients that underwent lumbar spine surgery for their presenting symptoms but only experienced improvement after undergoing cervical spine decompression. Overall, the patients that underwent a cervical operation experienced a dramatic decrease or complete resolution of their preoperative low back pain and radiculopathy (mean preoperative VAS of 6.7 vs. 3.7 postoperative).

Convincing a patient with mainly low back pain that a cervical operation is required can be difficult; however, the patient must be educated regarding the importance of treating the cervical spine, particularly when there is evidence of myelopathy. Patients presenting with lumbar symptoms disproportionate to clinical and radiographic imaging merit further workup. The clinician must elicit a broader and more detailed history from patients in these situations. Specific questions and exam findings attempting to elicit other causes must be directed towards the patient that presents with low back pain. Older case series similarly reported an improvement in myeloradiculopathy and a decrease in severity of lower extremity symptoms in patients who underwent cervical procedures only [4,14-15]. As our understanding of spinopelvic parameters in relation to global spine alignment becomes better delineated, a more formal treatment algorithm may be possible [16-17]. In these clinical vignettes, cervical stenosis associated with myelopathy was found to be an underlying contributor to the chief complaints. Importantly, the presenting symptom of severe low back pain resolved after surgical decompression of the severe cervical stenosis. This observation demonstrates the need for high clinical suspicion in patients that have presented symptoms discordant with their radiographic workup. As clinicians, there is no substitute for a detailed history and thorough physical exam to correlate to findings on imaging. In addition, further research is warranted to investigate global spine pathology rather than reviewing the cervical or lumbar spine in isolation.

There are several potential limitations with this study, with the most significant being the low number of patients in this case series. However, this was expected due to the elusive nature of diagnosing TSS and the short time frame covered by this case series. This study was also completed in a retrospective manner, which can lead to unintentional biases. The VAS pain score used to rate patient symptoms is a subjective scale and may be inconsistent among patients. A more appropriate and widely used objective grading scale such as the Japanese Orthopedic Association (JOA) score is now being utilized in current cases to provide clinical measures. Furthermore, this study may be affected by many potential confounding variables, such as differences in the specific procedures performed, various causes of back pain, and level of patient effort in postoperative rehabilitation. Further investigation of tandem stenosis with a larger prospective study and more objective clinical outcomes is warranted.

## Conclusions

Although TSS is not uncommon, its presence in a subset of patients with isolated lumbar symptoms with disproportionate clinical and radiographic findings should not be overlooked. In patients with continued low back pain and radiculopathy after lumbar decompression procedures, further investigation of the cervical spine should be a consideration. Clinical judgment should be used in assessing patients for surgery, while deferring to initial cervical decompression in unclear cases. The six cases we presented demonstrate that cervical stenosis may contribute to patients presenting with chiefly lumbar symptoms. Furthermore, cervical decompression alone only may provide clinically significant relief of these lumbar symptoms.
